# Fn14-Fc suppresses germinal center formation and pathogenic B cells in a lupus mouse model via inhibition of the TWEAK/Fn14 Pathway

**DOI:** 10.1186/s12967-016-0846-4

**Published:** 2016-04-21

**Authors:** Hong-Ki Min, Sung-Min Kim, Jin-Sil Park, Jae-Kyeong Byun, Jennifer Lee, Seung-Ki Kwok, Young-Woo Park, Mi-La Cho, Sung-Hwan Park

**Affiliations:** Rheumatism Research Center, Catholic Institutes of Medical Science, The Catholic University of Korea, 222 Banpo-Daero, Seocho-gu, Seoul, 137-701 South Korea; Division of Rheumatology, Department of Internal Medicine, College of Medicine, Seoul St. Mary’s Hospital, The Catholic University of Korea, 222 Banpo-Daero, Seocho-gu, Seoul, 137-701 South Korea; Integrative Omics Research Center, Korea Research Institute of Bioscience and Biotechnology, Daejeon, 305-806 South Korea

**Keywords:** TNF-like weak inducer of apoptosis (TWEAK), Fibroblast growth factor-inducible 14 (Fn14), Systemic lupus erythematosus (SLE), Germinal center (GC), Follicular helper T (Tfh) cell

## Abstract

**Background:**

Systemic lupus erythematosus (SLE) is an autoimmune-mediated chronic inflammatory disease. Half of patients with SLE suffer from lupus nephritis, which is major cause of death in SLE. TNF-like weak inducer of apoptosis (TWEAK)/fibroblast growth factor-inducible 14 (Fn14) interactions mediate inflammatory responses that are linked to the pathogenesis of lupus nephritis. Blocking of the TWEAK/Fn14 pathway by Fn14-Fc was performed in a SLE mouse model and the likely therapeutic mechanisms were investigated.

**Methods:**

To investigate the impact of TWEAK on B cell differentiation in SLE, the levels of *AID*, *Blimp*-*1*, and *IRF4* messenger RNA were measured in CD19^+^ B cells extracted from the spleens of sanroque mice and cultured with TWEAK. To identify the therapeutic effects of Fn14-Fc in SLE, sanroque mice were treated with Fn14-Fc or a control-Fc for 3 weeks. Immunoglobulin (Ig) G, IgG1, IgG2a, and anti-dsDNA antibody (Ab) levels were measured in the sera of each group. Spleens from each group were stained with antibodies against CD4, B220, GL-7, CD138, and PD-1. Kidneys were stained with hematoxylin and eosin (H&E) and periodic acid-Schiff (PAS).

**Results:**

Administration of TWEAK increased the mRNA levels of *AID*, *Blimp*-*1*, and *IRF4*. Treatment with Fn14-Fc suppressed levels of IgG, IgG1, IgG2a, and anti-dsDNA Ab in sera and reduced numbers of B, plasma, and follicular helper T (Tfh) cells in spleens of sanroque mice. In addition, renal protective effects of Fn14-Fc were shown.

**Conclusion:**

Fn14-Fc had beneficial effects in a SLE mouse model by repressing B cells, plasma cells, Tfh, and renal damage. This suggested that Fn14-Fc represents a potential therapeutic agent for SLE.

## Background

Systemic lupus erythematosus (SLE) is a systemic autoimmune disease that can damage many organ systems [1]. Several heterogenic factors, including genetic, epigenetic, environmental, and immunoregulatory components, contribute to the development of SLE [1]. Aberrant immune reactions in SLE are mediated by autoantibodies (autoAbs), immune complexes and autoreactive inflammatory cells, which result in organ damage. Females of childbearing age constitute the majority of SLE patients, and approximately half of SLE patients have kidney involvement. Lupus nephritis is a major cause of mortality and morbidity in SLE [2].

Autoantibodies and immune complexes play a crucial role in tissue damage and inflammatory responses in SLE. Plasma cells derived from B cells produce antibodies (Abs). Germinal centers (GCs) are the secondary lymphoid tissue in which B cell selection, differentiation, and maturation occur. In the GC, follicular helper T (Tfh) cells play an important role in B cell selection and differentiation toward plasma cells [3]. In SLE patients, dysregulation of Tfh in the GC plays a crucial role in the expansion of self-reactive B cells and the production of autoAbs [4]. Roquin protein regulates the development of Tfh in the GC, and a mutation of the *sanroque* gene results in excessive formation of Tfh and GC [5]. Roquin^San/San^ mice were selected as a SLE model in this study because the sanroque gene mutation causes lupus-like features through regulating Tfh and GC.

TNF-like weak inducer of apoptosis (TWEAK) is a proinflammatory cytokine that mediates several cellular and inflammatory responses by binding to fibroblast growth factor-inducible 14 (Fn14, also known as the TWEAK receptor). Recently, a link has been identified between the pathogenesis of several autoimmune disorders including autoimmune encephalitis, rheumatoid arthritis, and SLE with the TWEAK/Fn14 pathway [6, 7]. Xia et al. [8] demonstrated that the TWEAK/Fn14 pathway has a crucial role in the pathogenesis of Ab-induced nephritis, and disrupting the TWEAK/Fn14 pathway is a potential treatment for Ab-induced nephritides, including lupus nephritis. Recent studies revealed that the TWEAK/Fn14 interaction has an important role in the pathogenesis of several SLE manifestations [7, 9]. The TWEAK/Fn14 pathway contributes to the pathogenesis of SLE by modulating the local environment of the target organ [7, 10]. However, the TWEAK/Fn14 pathway activates nuclear factor kappa-light-chain-enhancer of activated B cells (NF-κB) signaling and the dysregulation of NF-κB signaling can induce autoimmune disorders by altering B and T cell immunity [11]. Therefore, the TWEAK/Fn14 interaction may have systemic effects on the pathogenesis of SLE in addition to local pathological effects.

We hypothesized that blocking the TWEAK/Fn14 pathway via administration of Fn14-Fc would attenuate the autoimmune response in a mouse model of SLE. To identify the mechanisms involved, we explored the effects of Fn14-Fc on Ab secretion, B cell maturation, Tfh cell development, GC formation and kidney damage. In addition, the pathologic role of TWEAK was investigated in sanroque mice by administration of TWEAK to B cells.

## Methods

### Animals

Roquin^san/san^ (sanroque) mice in a C57BL/6 background were obtained from the National Institutes of Health (Bethesda, MD, USA). The mice were maintained under specific pathogen-free conditions at the Catholic Research Institute of Medical Science at the Catholic University of Korea and were fed standard mouse chow (Ralston Purina, St. Louis, MO, USA) and water ad libitum. All experimental procedures were examined and approved by the Animal Research Ethics Committee of the Catholic University of Korea; the procedures conformed to all the USA National Institutes of Health guidelines.

### Preparation of Fn14-Fc

The Fn14-Fc and control-Fc used in the experiments (the hinge-CH_2_-CH_3_ form of IgG1) were bought from A&RT Therapeutics (Daejeon, South Korea).

### Murine B cell isolation and stimulation

Spleen cells were washed with phosphate-buffered saline (PBS; pH 7.2). After centrifugation at 1300 rpm and at 4 °C, the cells were incubated with CD19-coated magnetic beads (Miltenyi Biotec, Bergisch Gladbach, Germany) and isolated on MACS separation columns (Miltenyi Biotec). Positively selected CD19^+^ B cells were stimulated with TWEAK (0.1 ng/ml; R&D Systems, Minneapolis, MN, USA) for 3 days. Total RNA was extracted using the TRI Reagent (Molecular Research Center, Cincinnati, OH, USA).

### Treatment with Fn14-Fc

To assess the influence of Fn14-Fc on the severity of symptoms in the SLE model, sanroque mice were treated with 100 μg/mouse Fn14-Fc in saline or control-Fc via intraperitoneal injections three times weekly for 3 weeks. Treatment was started in 12-week-old sanroque mice. The groups were sacrificed 21 days after the first injection and the spleen and kidney were obtained at the time of sacrifice.

### Measurement of immunoglobulin (Ig) G subtypes and autoAbs

Blood was obtained from the orbital sinus of Fn14-Fc and control-Fc-treated mice and the serum was stored at −20 °C until use. Total IgG, IgG1, IgG2a, and anti-double-stranded (ds) DNA Abs were measured using a mouse total IgG, IgG1 and IgG2a enzyme-linked immunosorbent assay (ELISA) quantitation kit (Bethyl Laboratories, Montgomery, TX, USA). Anti-dsDNA was measured using double-stranded DNA–cellulose from calf thymus (Sigma, St. Louis, MO, USA) and an ELISA quantitation kit. Levels of total IgG, IgG1 and IgG2a were measured in mouse serum diluted 50,000-fold and the anti-dsDNA Ab was diluted 10-fold. The optical density (OD) at a wavelength of 450 nm of each well was measured using an ELISA plate reader (Bio-Rad, Hercules, CA, USA).

### Confocal microscopy

Spleen tissues were snap-frozen in liquid nitrogen and stored at −70 °C. Spleen tissue Sections (7 μm) were fixed in acetone and stained with Percp-Cy5.5 conjugated anti-CD4, APC-conjugated anti-B220, PE conjugated anti-PD-1 and anti-hu/mo CD266 (TWEAKR) (eBioscience, San Diego, CA, USA), FITC-conjugated anti-GL-7 and PE-conjugated anti-CD138 (BD Biosciences, San Jose, CA, USA). Stained sections were visualized using a Zeiss microscope (LSM 510 Meta; Carl Zeiss, Oberkochen, Germany) at ×400 magnification. The areas of GC and Tfh were analyzed per ×400 field on each section using ZEN 2009 Light Edition software. To eliminate sectioning artifacts, sections that were five slices apart were analyzed in each group.

### Histopathological and morphometric analysis

Mice were euthanized and their kidneys were harvested, fixed in 10 % buffered formalin, processed for paraffin embedding, cut into 5-µm tissue sections, and stained with hematoxylin and eosin (H&E) and periodic acid-Schiff (PAS). Tubular injury and glomerular matrix expansion was scored by a blinded assessor on 20 random glomeruli from each mice. The following scoring system was used: 0 (none), 1 (<25 % glomerular area involved), 2 (25–50 %), 3 (50–75 %), 4 (75–90 %), and 5 (>90 %).

### Real-time polymerase chain reaction (PCR)

Messenger RNA (mRNA) was extracted using TRI reagent (Molecular Research Center), according to the manufacturer’s instructions. Complementary DNA was synthesized using the SuperScript reverse transcription system (Takara, Otsu, Japan). A Light-Cycler 2.0 (software ver. 4.0; Roche Diagnostics, Basel, Switzerland) was used for the PCR amplification. All reactions were performed with a LightCycler FastStart DNA Master SYBR Green I kit (Takara), according to the manufacturer’s instructions. The following primers were used: activation-induced deaminase (AID), 5′-GCC ACC TTC GCA ACA AGT CT-3′ (sense), 5′-CCG GGC ACA GTC ATA GCA C-3′ (antisense); X-box binding protein 1 (xbp-1), 5′-GAT CCT GAC GAC GTT CCA GA-3′ (sense), 5′-ACA GGG TCC AAC TTG TCC AG-3′ (antisense); B lymphocyte-induced maturation protein-1 (Blimp-1), 5′-CTG TCA GAA CGG GAT GAA CA-3′ (sense), 5′-TGG GGA CAC TCT TTG GGT AG-3′ (antisense); interferon regulatory factor-4 (IRF4), 5′-GCA GCT CAC TTT GGA TGA CA-3′ (sense), 5′-AGG CCA AAC GTC ACA GGA CAT TG-3′ (antisense); β-actin, 5′-GTA CGA CCA GAG GCA TAC AGG-3′ (sense), 5′-GAT GAC GAT ATC GCT GCG CTG-3′ (antisense), Tnfrsf 12a (Fn14), 5′-GGC GCT GGT TTC TAG TTT CCT-3′ (sense), 5′-CAG TCT CCT CTA TGG GGG TAG T-3′ (antisense) and designed via using Primer Express (Applied Biosystems, Foster City, CA). mRNA levels were normalized to that of β-actin.

### Statistical analysis

The statistical analyses were performed using GraphPad Prism (ver. 5 for Windows; GraphPad Software). Numerical data were compared between the two groups using Student’s *t*-tests. A value of *P* < 0.05 was taken to indicate statistical significance.

## Results

### TWEAK promotes B cell differentiation in sanroque mice

**Fig. 1:**
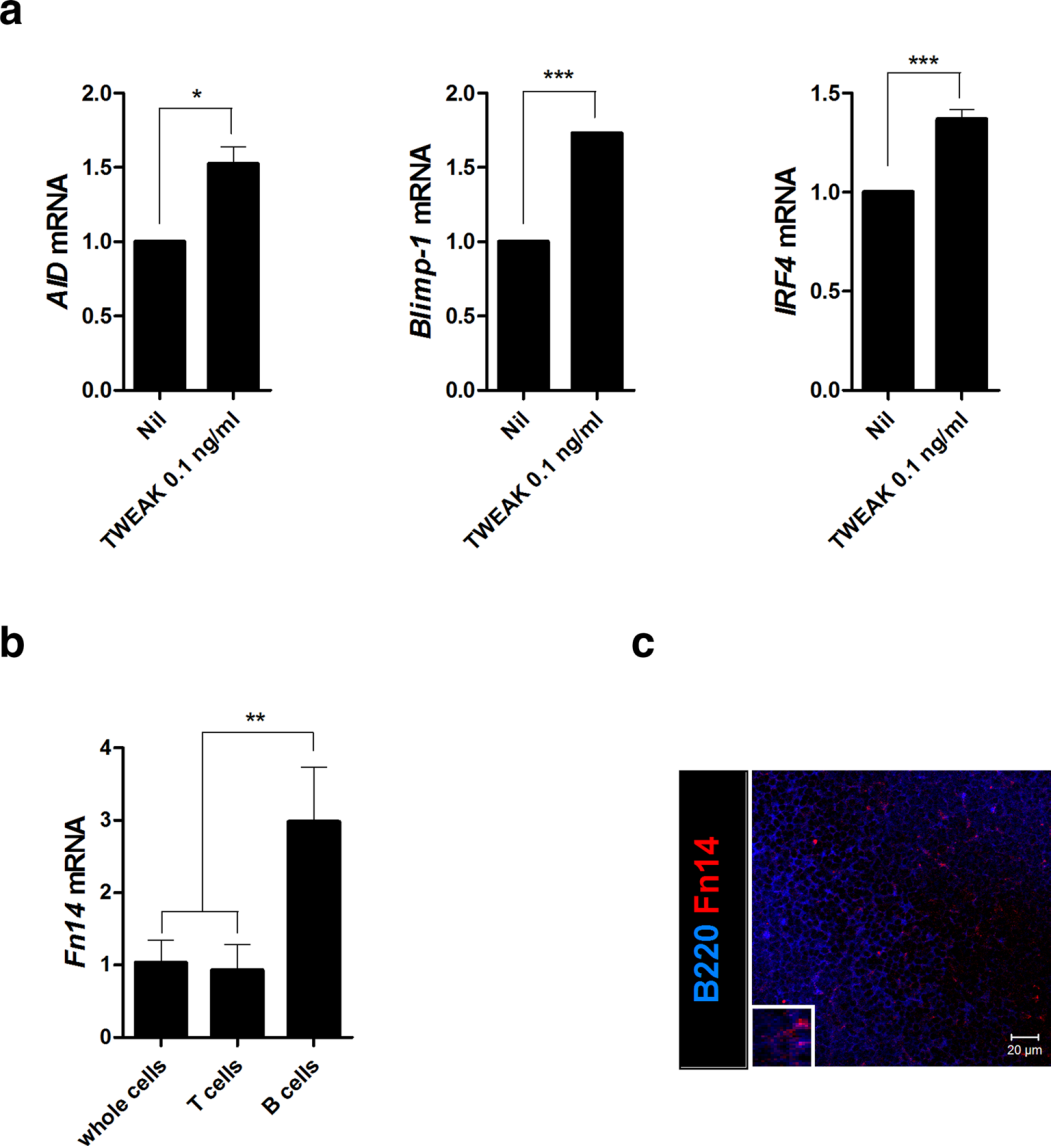
TWEAK promotes B cell differentiation. **a** CD19^+^ B cells isolated from the spleens of sanroque mice were cultured with or without TWEAK (0.1 ng/ml). After 3 days, total RNA was extracted using the TRI reagent. The mRNA levels of various B cell differentiation markers—such as *AID*, *Blimp*-*1*, *IRF4*—were analyzed by real-time PCR. **b** Splenocytes, CD4^+^ T cells, and CD19^+^ B cells were isolated from the spleens of sanroque mice, total RNA was extracted using TRI reagent, and mRNA levels of Fn14 were analyzed by real-time PCR. **c** Spleens from the sanroque mice were examined by immunofluorescence staining with monoclonal Abs against B220 (*blue*) and Fn14 (*red*). Data are expressed as means ± SDs. *P < 0.05, ***P < 0.001 compared to the control group

To assess the effect of TWEAK on B cell differentiation, CD19^+^ cells were extracted from the spleens of sanroque mice and cultured with or without TWEAK (0.1 ng/ml). After 3 days, total RNA was extracted from the cultured cells. In the TWEAK-administrated group, expression levels of B cell maturation and differentiation-associated genes, including *AID*, *Blimp*-*1*, and *IRF4* were significantly higher than in the control group (Fig. 1a). These results showed that TWEAK promoted B cell differentiation in SLE mouse models and that TWEAK could play an important role in aberrant humoral immunity in SLE. Fn14 expression was assessed by real-time PCR and confocal staining in the spleen. The level of *Fn14* mRNA was significantly higher in B cells than whole splenocytes and T cells (Fig. 1b). In addition, Fn14 expression on spleen B cells was confirmed by confocal staining (Fig. 1c).

### Suppression of Ig production, GC formation, and B cell differentiation in sanroque mice by Fn14-Fc

**Fig. 2:**
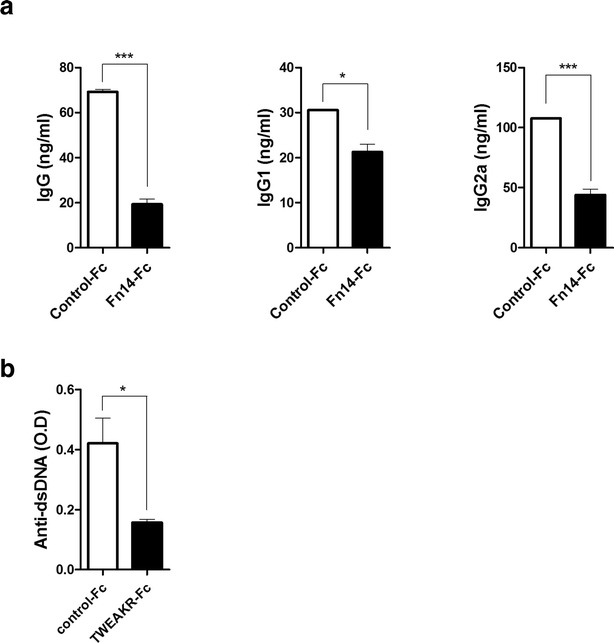
In vivo effect of Fn14-Fc on Ig production in SLE mice. Sanroque mice were injected intraperitoneally with Fn14-Fc (100 μg/mouse) or control-Fc (100 μg/mouse) (n = 5/group) for 3 weeks. Mice were sacrificed on day 21 after the first injection. The serum total IgG, IgG1 and IgG2a levels were determined by ELISA. Data are expressed as means ± SDs. *P < 0.05, **P < 0.01 compared to the control-Fc treated group

Sanroque mice were treated with Fn14-Fc or control-Fc (100 μg/mouse) three times per week for 3 weeks by intraperitoneal injection. Mice were sacrificed 21 days after the first injection. Levels of Ig were measured in the sera of each group. The IgG, IgG1, IgG2a, and anti-dsDNA levels of the Fn14-Fc-treated group were significantly lower than those of the control-Fc-treated group (Fig. 2a, b).

**Fig. 3:**
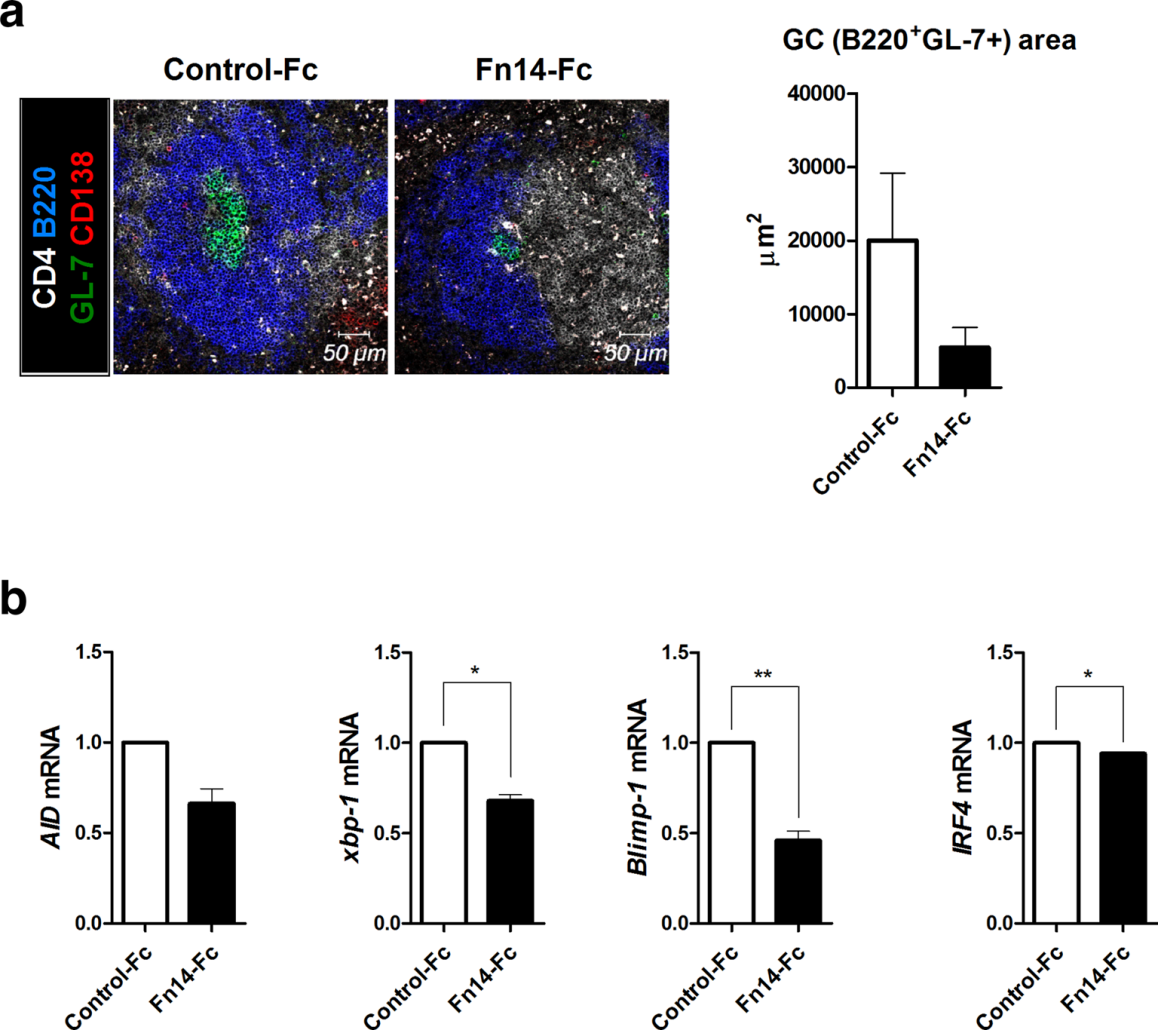
Effects of Fn14-Fc on GC formation and B cell transcription factors in SLE mice. **a** Fn14-Fc or control-Fc-treated mice were sacrificed 21 days after the first injection. Spleens from the mice in each group (n = 5/group) were examined by immunofluorescence staining with monoclonal Abs against CD4 (*white*) and B220 (*blue*), GL-7 (*green*) and CD138 (*red*) (original magnification, ×400). **b** mRNA levels of *AID*, *xbp*-*1*, *Blimp*-*1* and *IRF4* in isolated splenocytes were determined by real-time PCR. Data are expressed as means ± SDs. *P < 0.05, **P < 0.01 compared to the control-Fc treated group

Spleens extracted from each group were stained with Abs against CD4 (white), B220 (blue), GL-7 (green), and CD138 (red). mRNA levels were determined by real-time PCR of the splenocytes from each group. Spleens from Fn14-Fc-treated mice demonstrated reduced numbers of B220^+^, GL-7^+^, and CD138^+^ cells, but not of CD4^+^ cells (Fig. 3a). Fn14-Fc administration significantly reduced the mRNA levels of *xbp*-*1*, *Blimp*-*1*, and *IRF4*. Expression of *AID* mRNA was suppressed in the Fn14-Fc group, but the difference was not statistically significant (Fig. 3b). Fn14-Fc efficiently repressed Ab production, GC formation, and differentiation of B cells in sanroque mice.

### Fn14-Fc reduces the Tfh cell population in the spleens of sanroque mice

**Fig. 4:**
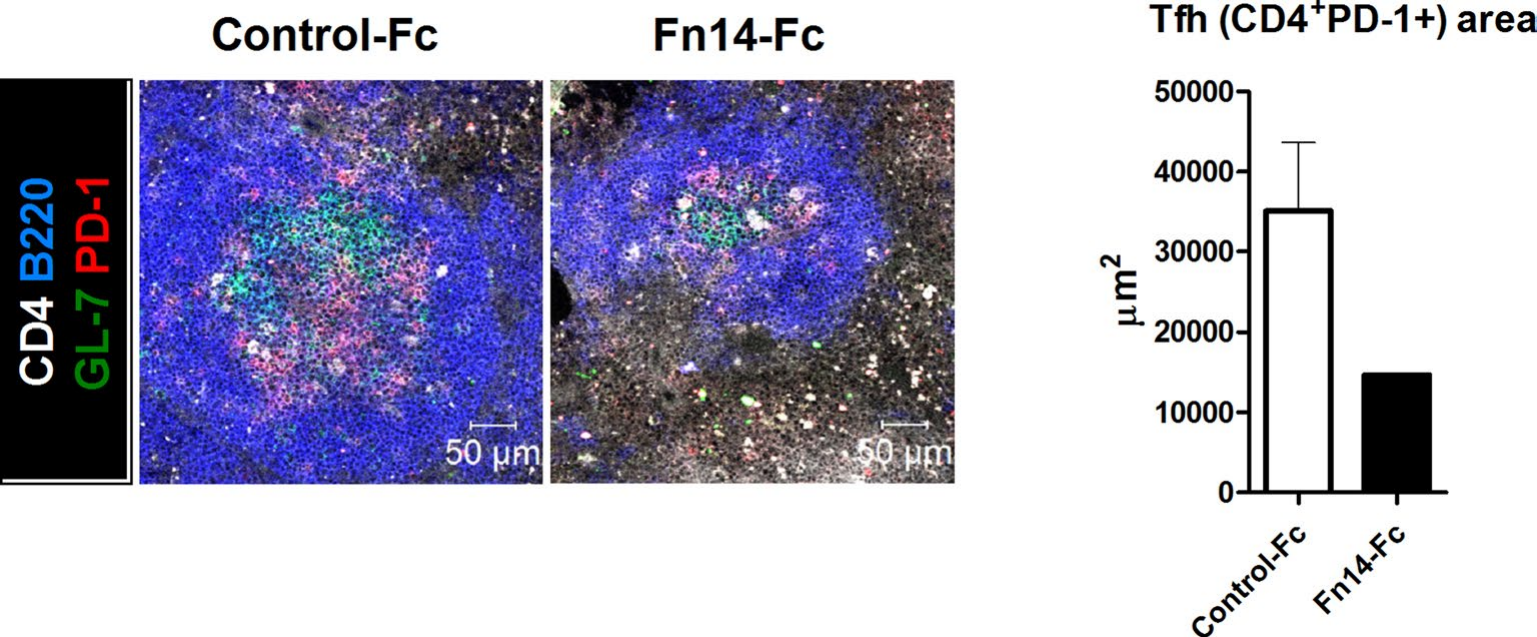
Fn14-Fc reduced Tfh cell populations. Fn14-Fc or control-Fc-treated mice were sacrificed 21 days after the first injection. Spleens from mice in each group (n = 5/group) were examined by immunofluorescence staining with monoclonal Abs against CD4 (*white*), B220 (*blue*), GL-7 (*green*), and PD-1 (*red*). Original magnification ×400

Sanroque mice were treated with Fn14-Fc or control-Fc and sacrificed as above. The spleens from both groups were stained with Abs against CD4 (white), B220 (blue), GL-7 (green), and PD-1 (red). Immunofluorescence staining showed a reduction in the numbers of B220^+^, GL-7^+^, and PD-1^+^ cells in Fn14-Fc-treated mice compared to control-Fc-treated mice (Fig. 4). This finding suggested that Fn14-Fc could suppress not only B cell formation but also Tfh cell differentiation in GC.

### Fn14-Fc improves nephritis in SLE mice

**Fig. 5:**
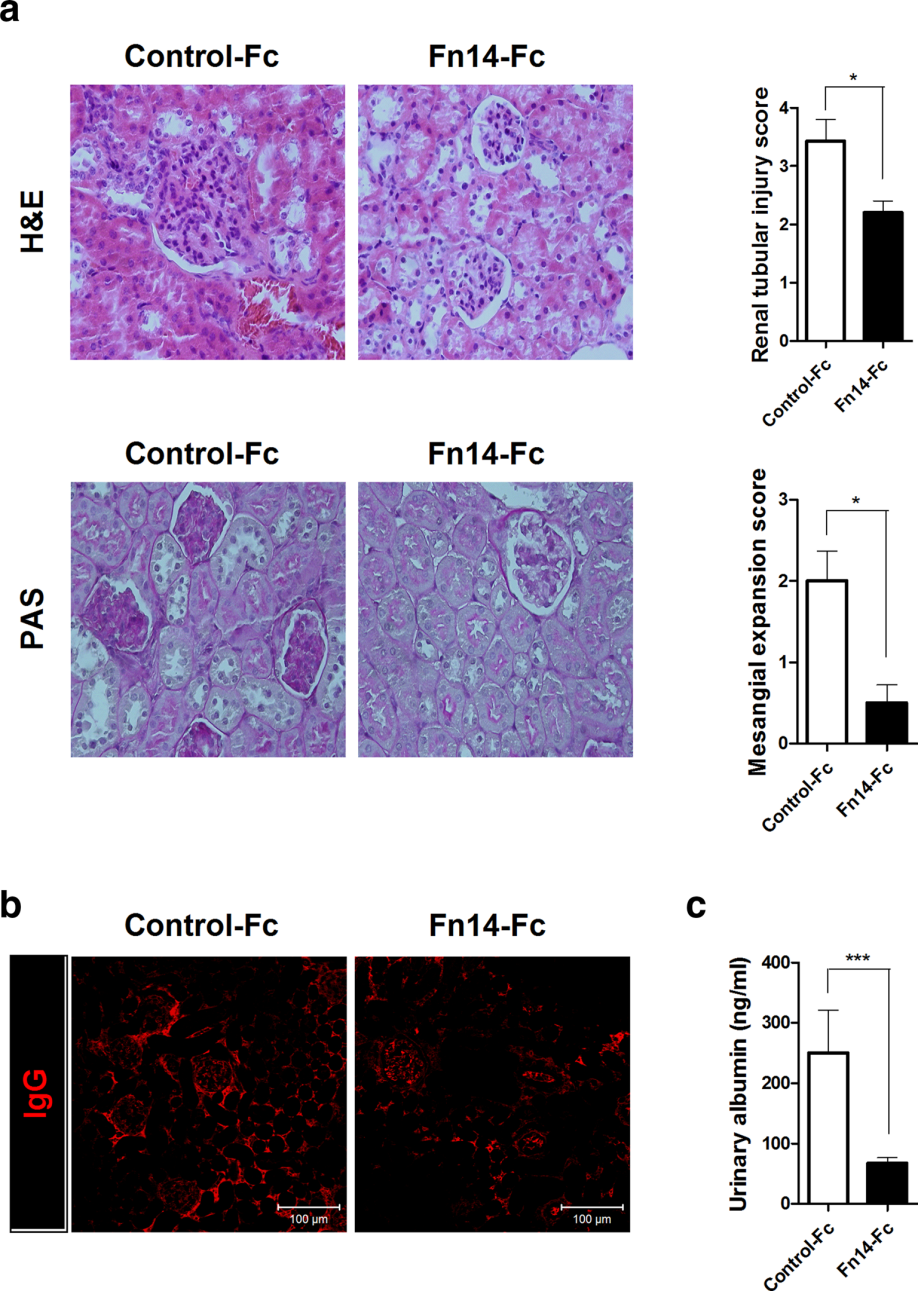
Fn14-Fc improved nephritis in SLE mice. Sanroque mice were injected intraperitoneally with Fn14-Fc (100 μg/mouse) or control-Fc (100 μg/mouse) (n = 5/group) for 3 weeks. Mice were sacrificed on day 21 after the first injection. Sections of kidney tissue were stained with H&E (**a**) and PAS (**b**). Original magnification ×400

The kidneys were extracted from sanroque mice treated with Fn14-Fc or control-Fc for 3 weeks (with a treatment schedule and sacrifice protocol performed as described above). The kidneys were stained with H&E and PAS. The structures of the glomeruli and tubules were preserved in the Fn14-Fc group, whereas the control-Fc group showed deterioration (Fig. 5a). IgG deposition and urinary albumin excretion were significantly lower in the Fn14-Fc group than in the control-Fc group (Fig. 5b, c). These differences indicate that Fn14-Fc has beneficial effects on nephritis in SLE mouse models, which spontaneously developed glomerulonephritis and renal tubular damage.

## Discussion

The results of this study showed that blocking the TWEAK/Fn14 pathway using Fn14-Fc showed therapeutic effects in a SLE mouse model. The therapeutic effects could be explained by three mechanisms; the major mechanism was suppression of B cell differentiation and GC formation; inhibition of Tfh cell proliferation; and prevention of renal damage in a SLE mouse model. Additionally, a novel pathological role for TWEAK on SLE in which TWEAK promoted B cell maturation was revealed.

TWEAK belongs to the TNF ligand family and is a proinflammatory cytokine [12]. Dysregulated and overactivated TWEAK/Fn14 interactions induced inflammatory responses and tissue remodeling, which could induce autoimmune and chronic inflammatory disease [13, 14]. Fn14 is expressed in many tissues, but especially in the kidneys. TWEAK/Fn14 interactions play an important pathogenic role in Ab-induced nephritis in a mouse model [8]. Recent studies showed an association between the activities of lupus nephritis and levels of urinary TWEAK, and suggested that urinary TWEAK could act as a biomarker for lupus nephritis [15–17]. Furthermore, administration of TWEAK to human kidney cells induced processes featured in glomerulonephritis by promoting expression of inflammatory mediators, infiltration of inflammatory cells and proliferation of kidney cells [18]. The TWEAK/Fn14 pathway represents a potential therapeutic target for lupus nephritis [19, 20]. This study examined the renoprotective effects of Fn14-Fc in a SLE mouse model.

The relationship between SLE pathogenesis and the TWEAK/Fn14 pathway has been the subject of much discussion in the fields of lupus nephritis, neuropsychiatric lupus, and vascular injury in SLE [19]. A deficiency of TWEAK induced by mutation resulted in Ab deficiency, as reported in a recent case that suggested that TWEAK regulates B cell differentiation by controlling BAFF activation [21]. We examined the influence of TWEAK on BAFF, and confirmed that *BAFF* mRNA was upregulated by TWEAK stimulation and reversed by a TWEAK-neutralizing monoclonal Ab (not shown). However, these data are insufficient to prove that TWEAK stimulates B cell differentiation solely by affecting BAFF activation. To date, the pathologic effects of TWEAK on the maturation and function of B cells have not been fully explained. In this study, administration of TWEAK to splenic CD19^+^ cells from sanroque mice increased the expression of B cell-associated genes, including *AID*, *Blimp*-*1*, and *IRF4.* Each of these genes is critical for B cell differentiation and functions, including maturation, Ab diversification, plasma cell generation, and GC formation. We showed that Fn14 is highly expressed by B cells, and this suggests that TWEAK controls the proliferation of B cells, GC, and Tfh in SLE pathogenesis by directly affecting B cells via the TWEAK/Fn14 interaction. Further studies are needed to clarify the mechanisms of TWEAK in SLE pathogenesis. Nevertheless, this study is the first to clarify the pathogenic role of TWEAK in B cells by up-regulation of associated genes and to show Fn14 expression on B cells.

Circulating autoAbs and immune complexes are a pathologic factor of SLE and play a critical role in its pathogenesis. Pathogenic autoAbs are produced from auto-reactive plasma cells differentiated from B cells of GC. Tfh cells have been identified as a subtype of CD4^+^ T cells distinct from type 1 helper T cells (Th1) and Th2. The majority of Tfh cell resides in the GC and play important roles in GC-induction, selection of B cells in the GC, generation of memory B cells and long-lived plasma cells from selected B cells, and the production of high-affinity Abs [22]. Tfh cells are characterized by expression of PD-1, CXCR5, SAP, and ICOS on the cell surface [23]. The aforementioned roles of Tfh cell suggest that aberrant Tfh in autoimmune disease selects auto-reactive B cells to mature in GC and become plasma cells, which then produce high-affinity pathogenic autoAbs [24]. Furthermore, inhibition of aberrant Tfh cell functions has potential as a new therapeutic target for SLE [4]. In this study, Fn14-Fc was shown to suppress Tfh cell proliferation in vivo in sanroque mice.

In present study, we could show that blocking TWEAK/Fn14 signaling by Fn14-Fc suppressed B cell and Tfh cell activation and eventually manifestation of SLE in sanroque mice. It is not clear that which factor (B cell maturation versus Tfh cell differentiation) is more important on beneficial effects of Fn14-Fc in SLE mice. Futher study of TWEAK/Fn14 signaling on SLE could clarify aforementioned issue.

## Conclusions

In conclusion, Fn14-Fc had therapeutic effects in the sanroque mouse SLE model. Fn14-Fc suppressed the development of the GC, B cells, plasma cells, and Tfh cells. As a result, Ig secretion was repressed in Fn14-Fc treated sanroque mice. Additionally, glomerulonephritis and renal tubular damage in sanroque mice was improved by Fn14-Fc treatment. These results are worthy because they demonstrate for the first time that blocking the TWEAK/Fn14 pathway regulates systemic autoimmunity in SLE mice. Fn14-Fc treatment represents a potential therapeutic option in SLE, and especially in lupus nephritis.
